# Characterization of a new bifunctional endo-1,4-β-xylanase/esterase found in the rumen metagenome

**DOI:** 10.1038/s41598-021-89916-8

**Published:** 2021-05-17

**Authors:** Gabriella Cavazzini Pavarina, Eliana Gertrudes de Macedo Lemos, Natália Sarmanho Monteiro Lima, João Martins Pizauro Jr.

**Affiliations:** 1 Technology Department, School of Agricultural and Veterinarian Sciencess, Sao Paulo State University (Unesp), Via de Acesso Prof. Paulo Donato Castellane S/N, km 5, Sao Paulo, Brazil; 2 Graduate Program in Agricultural and Livestock Microbiology, School of Agricultural and Veterinarian Sciences, Sao Paulo State University (Unesp), Jaboticabal, Sao Paulo Brazil; 3 Molecular Biology Laboratory, Bioenergy Research Institute (IPBEN), Jaboticabal, Sao Paulo Brazil

**Keywords:** Enzymes, Metagenomics

## Abstract

Metagenomic data mining of the *Nellore cattle* rumen microbiota identified a new bifunctional enzyme, endo-1,4-β-xylanase/esterase, which was subsequently overexpressed in *E. coli* BL21 (DE3). This enzyme was stable at pH intervals of 5 to 6.5 and temperatures between 30 and 45 °C, and under the test conditions, it had a V_max_ of 30.959 ± 2.334 µmol/min/mg, K_m_ of 3.6 ± 0.6 mM and k_cat_ of 2.323 ± 175 s^−1^. Additionally, the results showed that the enzyme is tolerant to NaCl and organic solvents and therefore is suitable for industrial environments. Xylanases are widely applicable, and the synergistic activity of endo-1,4-β-xylanase/esterase in a single molecule will improve the degradation efficiency of heteroxylans via the creation of xylanase binding sites. Therefore, this new molecule has the potential for use in lignocellulosic biomass processing and as an animal feed food additive and could improve xylooligosaccharide production efficiency.

## Introduction

Lignocellulosic biomass is an important and strategically valuable source of renewable energy, and it has received considerable interest from investors due to its abundance as a raw material and potential use as a biofuel and for bioproduct production^1^. This biomass is composed of high levels of cellulose, hemicellulose and lignin bound by complex connections, which gives it native recalcitrance to enzymatic attacks, thus preventing its easy solubilization and utilization^2^.

Xylan is the most abundant hemicellulose in nature and contains mainly β-D-xylopyranosyl residues linked by β (1 → 4) glycosidic bonds. Some xylans have L-arabinofuranosyl and 4-*O*-methylglucuronic residues in their structure that are linked by α-1,2 and α-1,3 and present ester bonds with cumaric and ferulic acids^3^. Hemicellulose biodegradation, such as that occurring with xylans, involves the synergistic action of several hydrolytic enzymes (acetyl xylan esterase, α-D-glucoronidase and α-L-arabinofuranosidase), including main xylanolitic enzymes, such as endo-1,4-β-xylanases (EC 3.2.1.8) and β-xylosidases (EC 3.2.1.37)^4^. Endo-1,4-β-xylanases are responsible for random cleavage of the xylan skeleton at β glycosidic bonds (1 → 4)^3^, and in the Carbohydrate-Active Enzymes Database (CAZY, http://www.cazy.org/)^5^, they are mainly classified as belonging to the glycosyl hydrolases (GHs) group in the GH10 and GH11 families due to their amino acid sequence homology and catalytic domains^6^.

The conversion of lignocellulosic biomass is complex and requires treatments that can be physical, chemical, enzymatic or a combination of types^2,7^. Enzymatic treatments are environmentally safe and thus more interesting, thereby highlighting the need to characterize new enzymes and identify their functional diversity. Xylanases are widely used as hemicellulose-degrading enzymes and in processes such as saccharification, fruit juice processing, paper production^4^, second-generation ethanol production^3^, xylooligosaccharide production^8^ and xylitol acquisition^9^.

In the era of big data, the use of a metagenomic approach to query available databases can be valuable for mining and discovering new enzymes^10^, thus facilitating the exploration of the genetic diversity of environments, which cannot be achieved by conventional culture methods. The rumen is a highly efficient lignocellulose converting environment^11–13^, and metagenomic analyses of these structures could enable the identification of new carbohydrases, including xylanases. In this study, we performed metagenomic data mining of the rumen microbiota of *Nelore cattle* and identified the *xylr* gene, which is responsible for the production of a bifunctional xylanase esterase that is tolerant to salt and organic solvents. This gene was cloned into an expression vector and purified, and the enzyme was characterized.

## Results

### New xylanase gene prospected from the *Nelore cattle* rumen metagenome

**Figure 1:**
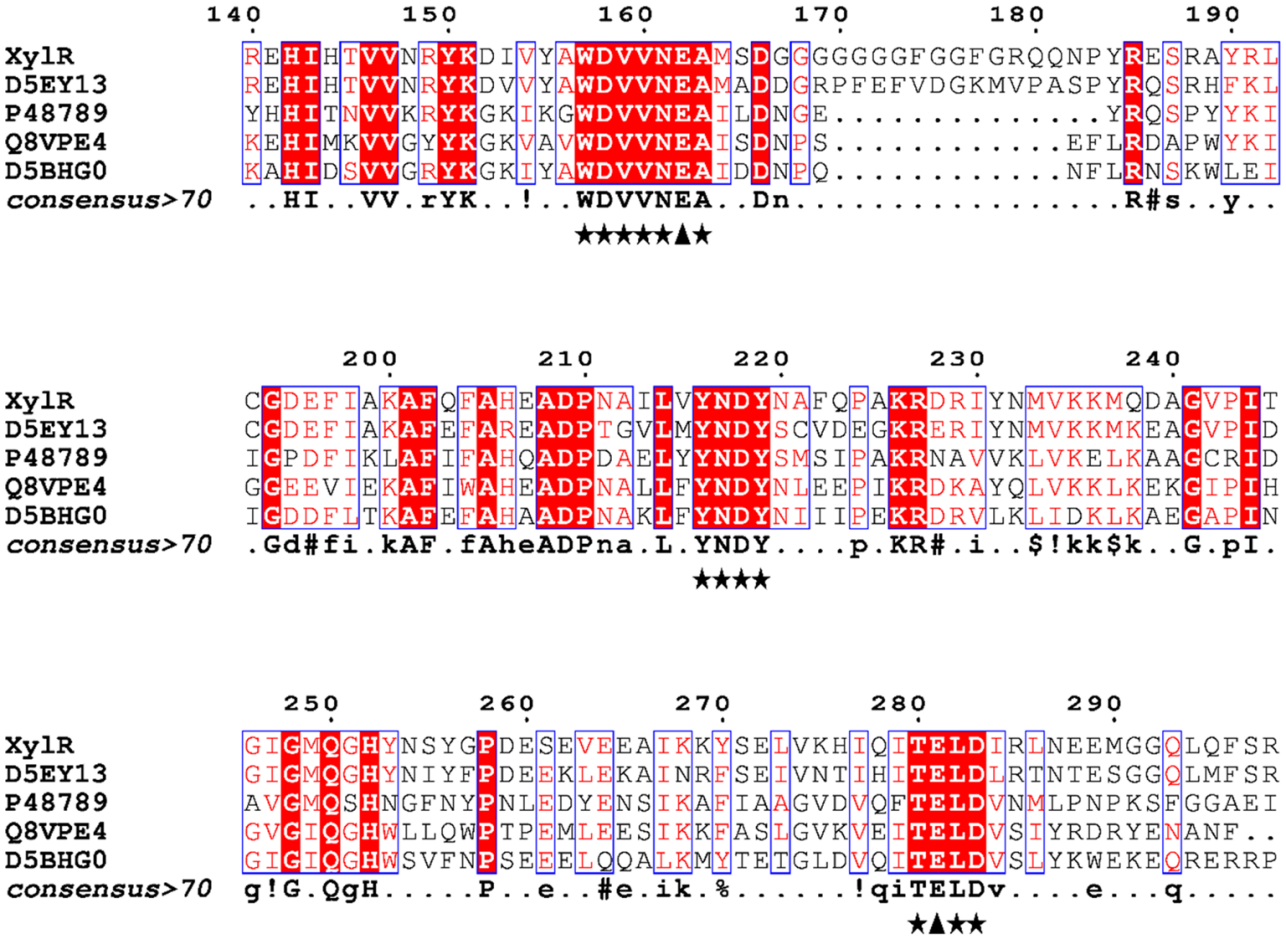
Fragment of the multiple alignment between the amino acid sequence of XylR and xylanases belonging to GH10. The conserved regions are marked with ★, and the conserved catalytic residues of glutamate (E) are highlighted with ▲. The alignment included xylanase sequences from Prevotella ruminicola (D5EY13), Prevotella ruminicola (P48789), Zunongwangia profunda (ADF53358) and a noncultivable bacterium (AAL06078). Similar amino acids are highlighted in red. Multiple alignment was performed using the Clustal Omega tool^16^, and assembly was performed in EsPript 3.0^17^.

**Figure 2:**
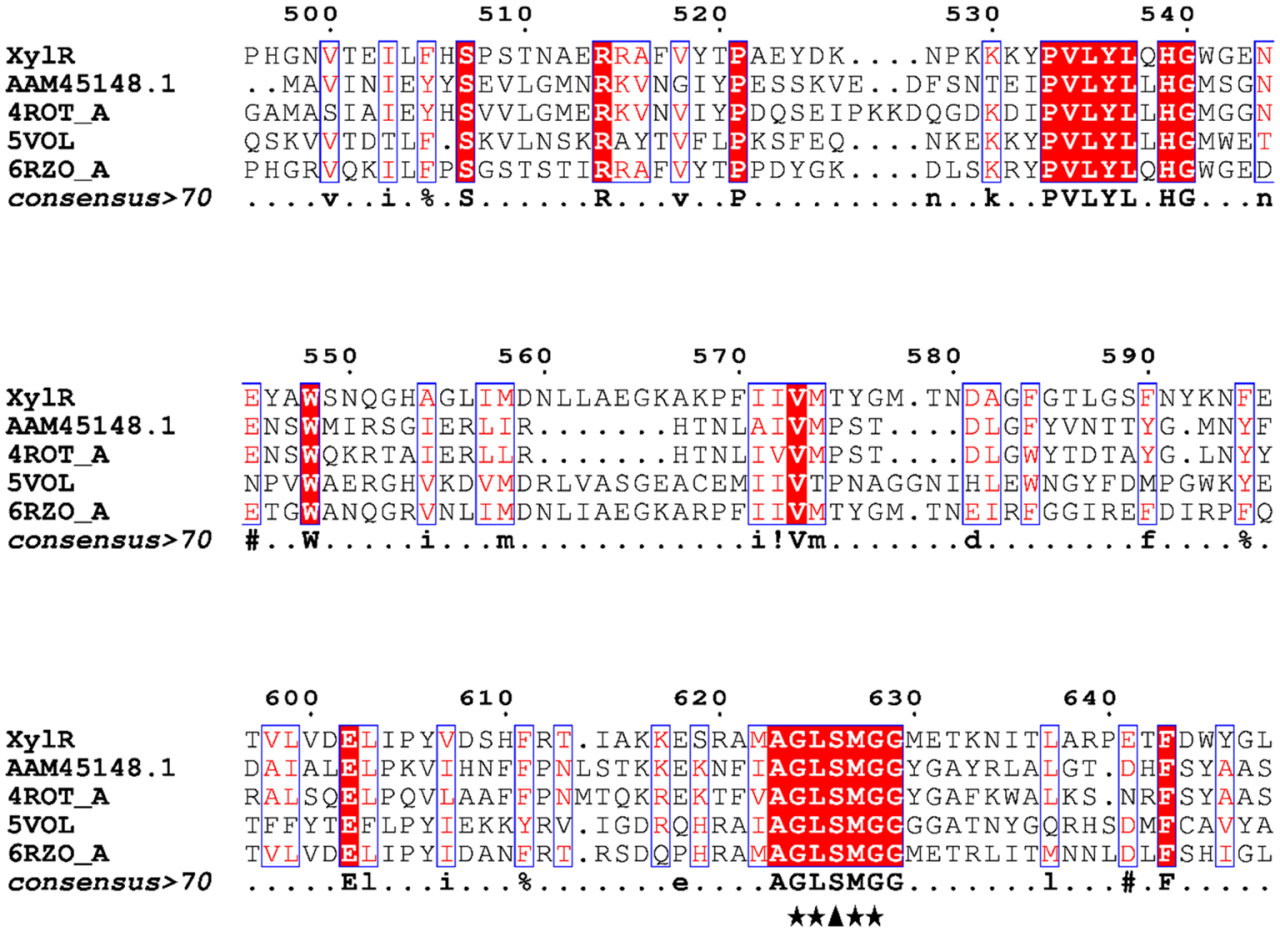
Fragment of the multiple alignment between the XylR amino acid sequence and CE1 family esterases. The conserved regions are marked with ★, and the conserved catalytic residue of serine (S) is highlighted with ▲. The alignment includes the esterase sequences of Lactococcus lactis (AAM45148), Bacteroides intestinalis DSM 17,393 (5 VOL), Streptococcus pyogenes (4ROT) and a noncultivable bacterium (6RZO). Similar amino acids are highlighted in red. Multiple alignments were performed using, the Clustal Omega tool^16^ and assembly was performed in EsPript 3.0^17^.

With the aid of a sequence-driven approach to evaluating the metagenomic data of the *Nelore cattle* rumen (SRX818104), the *xylr* gene encoding an endo-1,4-β-xylanase was identified. The *xylr* gene contains 2196 bp and 723 amino acids and has a signal peptide of 21 amino acid residues at the N-terminus with a predicted molecular mass of 81.6 kDa^14^. Using the BLASTp tool^15^, endo-1,4-β-xylanase was shown present 64% sequence similarity with the endo-1,4-β-xylanase/feruloyl esterase enzyme from *Prevotella ruminicola* (Table S1) and 66% similarity to a 2015 United States patent for hemicellulose-degrading enzymes (US 9012186). Multiple sequence alignments and secondary structure predictions demonstrated that the enzyme had the following conserved regions with xylanase GH10: ^159^WDVVNEA^163^ and ^280^TELD^283^ (Fig. 1). Regarding the esterase domain, the presence of the ^624^GLSMG^628^ motif with a serine (S) was observed at the center of this conserved region (Fig. 2).

### XylR was cloned, expressed in the soluble fraction and purified

The *xylr* gene was amplified directly from the rumen metagenomic DNA in the Laboratory of Biochemistry of Microorganisms and Plants (LBMP) database at São Paulo State University, Jaboticabal Campus. Thereafter, it was cloned and expressed in *E. coli* BL21 (DE3) (Figure S1) using the pET28a( +) vector. A fusion protein with His_6_ at the C-terminus was obtained. The cloning was confirmed by colony PCR and Sanger sequencing.

**Figure 3:**
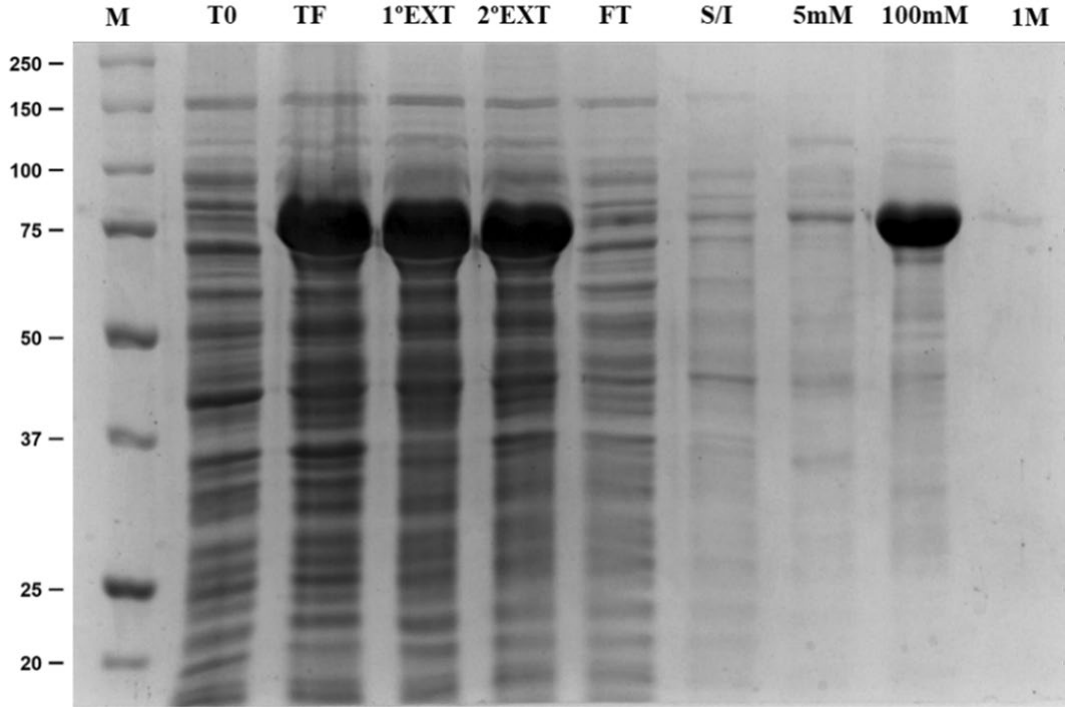
Electrophoretic profile of soluble proteins from *E. coli* BL21 that were transformed into the vector pET28a/ORF1374. Purification was performed by affinity chromatography on Ni–NTA resin. Channels: (M) Precision Plus marker; (T0) Time before induction; (TF) After 22 h of induction; (1°EXT) First extract; (2nd EXT) Second Extract; (FT) Flow through; (S/I) Fraction without imidazole; (5 mM) Fraction with the concentration of 5 mM imidazole; (100 mM) Fraction with 100 mM imidazole concentration; and (1 M) Fraction with a concentration of 1 M.

**Figure 4:**
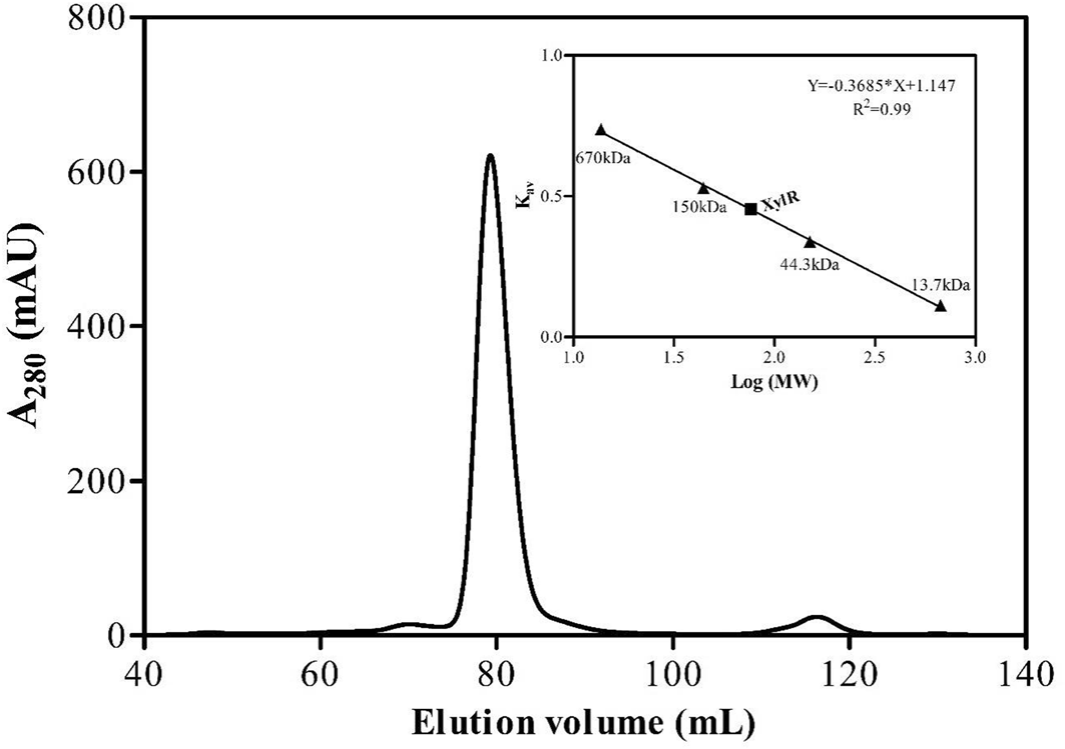
Chromatogram of purified XylR obtained by gel filtration using a Hiload 16/600 Superdex 200 column with a flow rate of 0.7 ml/minute. The internal image corresponds to the linear regression of the chromatography using the Protein Standard Mix 15 ± 600 kDa standard. Image built by GraphPad Prism software version 5.00. Image built by ImageLab 4.1 software. Notes: Protein Standard Mix 15 ± 600 kDa: Bovine thyroglobulin (670 kDa); bovine blood γ-globulin (150 kDa); chicken egg albumin fraction VI (44.3 kDa) and ribonuclease A (13.7 kDa).

The optimum conditions for the expression of the enzyme in *E. coli* BL21 (DE3) were obtained with a final concentration of IPTG of 0.1 mM and grown at 30 °C for 22 h at 180 rpm. Under these conditions, the recombinant protein was obtained from the cell lysate supernatant soluble fraction (Fig. 3). The XylR enzyme was purified by immobilized metal ion affinity chromatography (Fig. 3) and later by gel filtration chromatography (Fig. 4). SDS-PAGE analysis showed that the purified enzyme had a molecular weight of 75 kDa. The expression and purification of the recombinant enzyme was confirmed by western blot analysis with anti-His6 antibodies, which confirmed that the purified protein was expressed heterologously (Figure S2).

### XylR has a high affinity for xylan and short chain esters

**Figure 5:**
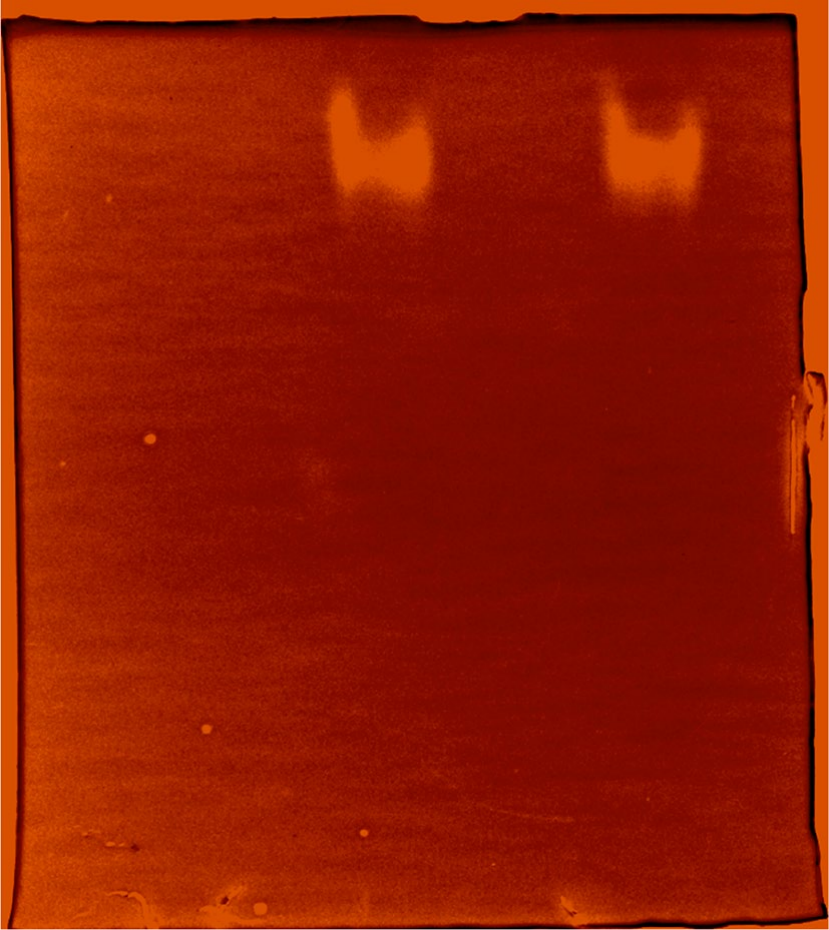
Zimogram for endo-1,4-β-xylanase activity obtained in a 6% polyacrylamide gel copolymerized with xylan from beechwood.

**Figure 6:**
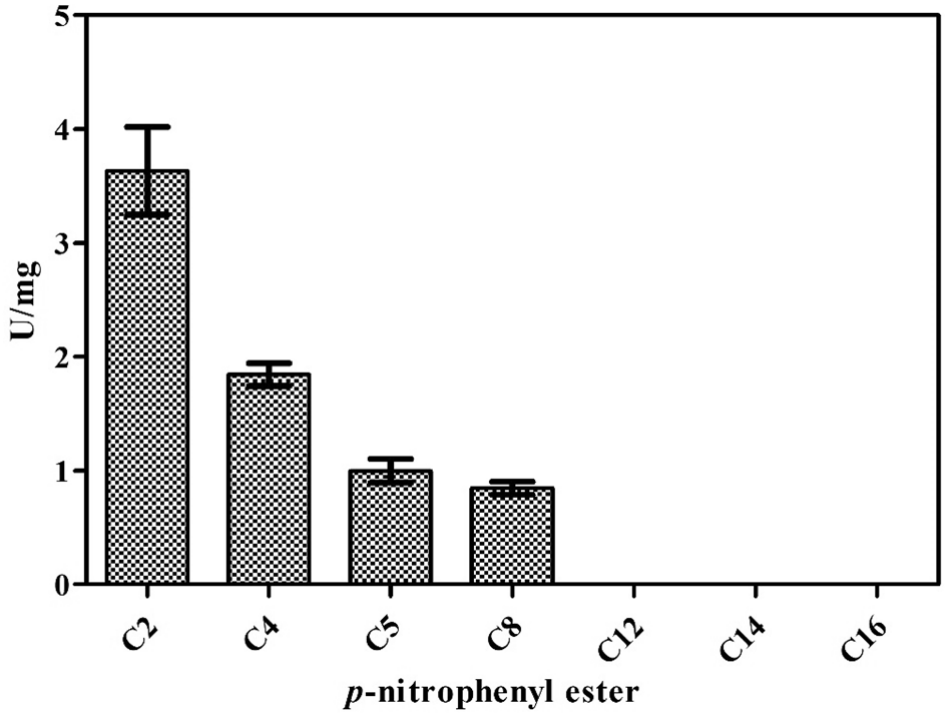
Affinity of XylR for esters of different carbons. Legend: (C2) *p*-nitrophenyl acetate, (C4) *p*-nitrophenyl butyrate, (C5) *p*-nitrofenyl valerate, (C8) *p*-nitrophenyl octanoate, (C12) *p*-nitrofenyl dodecanoate, (C14) *p*-nitrofenyl myristate, and (C16) *p*-nitrofenyl palmitate. Values are represented as the mean ± standard deviation.

The XylR enzyme has endo-1,4-β-xylanase (Fig. 5) and esterase activity for short chain esters (Fig. 6). These catalytic properties were determined by the kinetic constants (K_m_, V_max_, kcat and kcat/K_m_), with xylan from beechwood and p-nitrophenyl acetate used as substrates (Table 1). The comparative hypothesis test between the Michaelis–Menten kinetic model and the sigmoidal kinetic model demonstrated that the enzyme was more adjusted to the Michaelian model in relation to the substrates analyzed (xylan from beechwood p = 0.1; *p*-nitrophenyl acetate p = 0.5).

**Table 1:** Kinetic parameter values calculated for XylR. The values are represented as the mean ± standard deviation.

Substrates	V_max_ (µmol/min/mg)	kcat (s^−1^)	K_m_ (mM)	kcat/K_m_ (mM^−1^ s^−1^)	K_m_ (mg/ml)	kcat/K_m_ (mg ml^−1^ s^−1^)
Xylan from beechwood	30.959 ± 2.334	2.323 ± 175.1	3.2 ± 0.6	726.0	1.04 ± 0.18	2.23 × 10^3^
*p*-nitrofenyl acetate	66.0 ± 5.2	5,0 ± 0.4	2.3 ± 0.4	2.2	–	–

### XylR activity at neutral pH and room temperature

**Figure 7:**
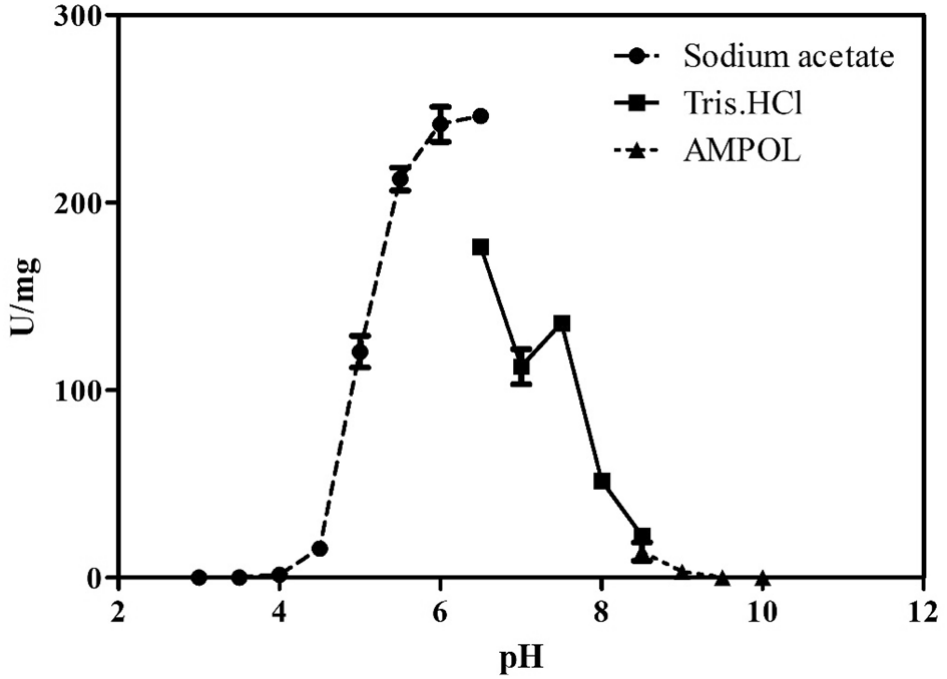
Relative activity at different pH values and ionic species. Values are represented as the mean ± standard deviation.

**Figure 8:**
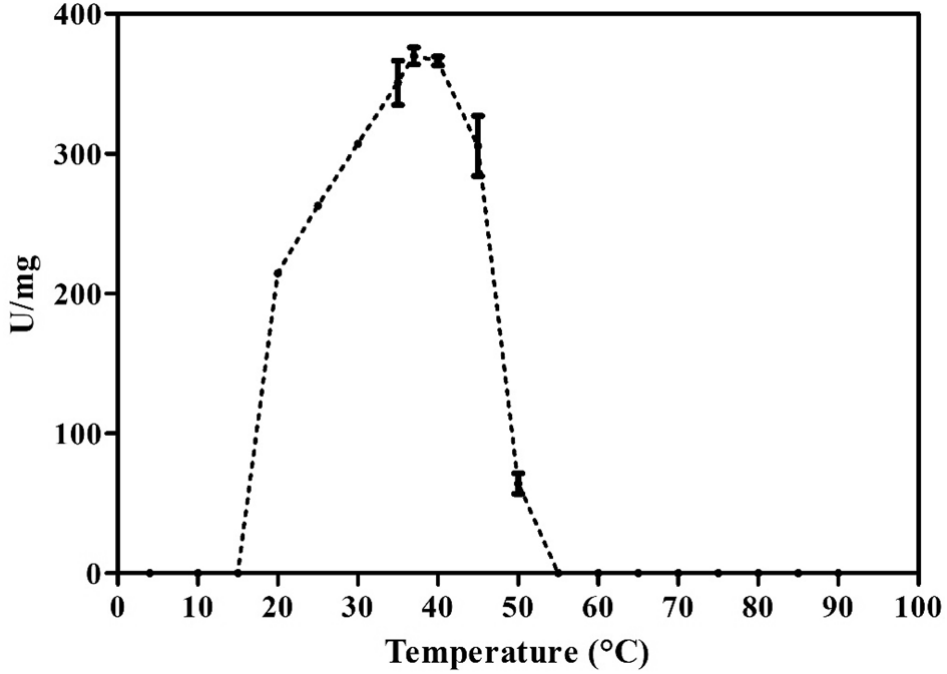
Relative activity at different temperatures. Values are represented as the mean ± standard deviation.

**Figure 9:**
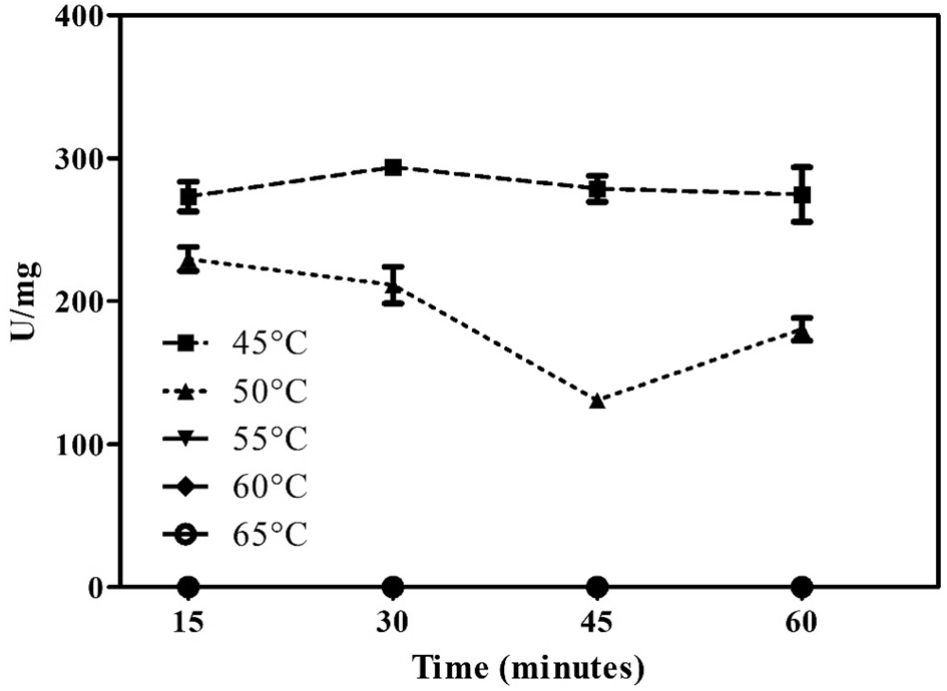
Enzyme stability when submitted to heat treatments of 45 and 50 °C for up to 60 min. Values are represented as the mean ± standard deviation.

Enzyme activity was observed with 0.1 M sodium acetate buffer at pH 5.5 to 6.5, and optimal hydrolysis occurred in this range (Fig. 7). The temperature effect showed that the optimal enzyme activity occurred at 37 °C and approximately 80% activity was maintained in intervals of 30 and 45 °C (Fig. 8). Additionally, the enzyme remained stable when subjected to heat treatments of 45 and 50 °C for up to one hour (Fig. 9).

### XylR is tolerant to high concentrations of NaCl

**Figure 10:**
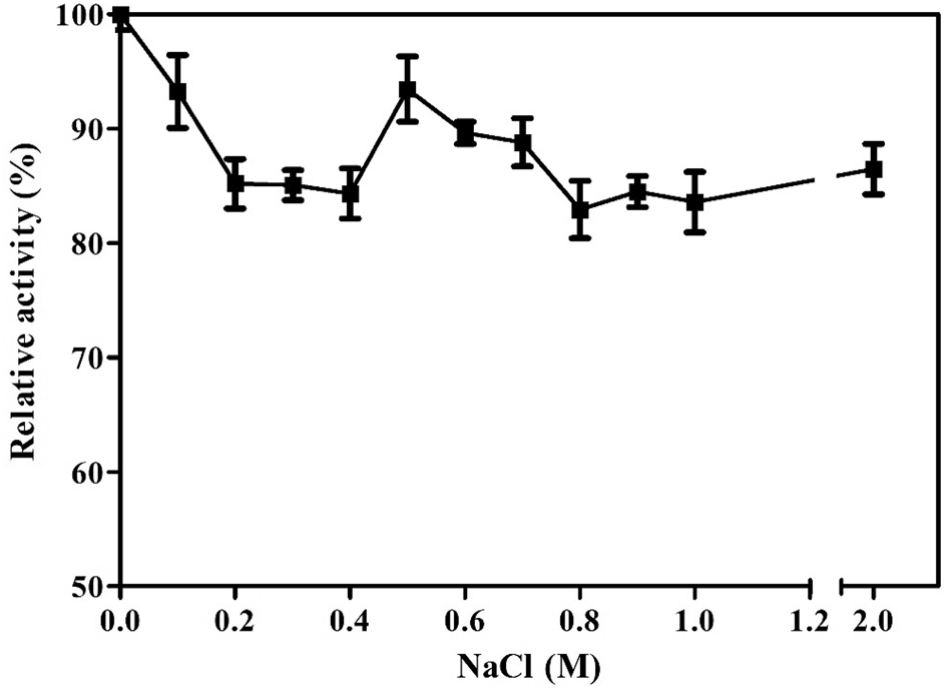
XylR activity in the presence of NaCl in the reaction medium at concentrations of 0.1 to 2 M. Values are represented as the mean percentage ± standard deviation.

**Figure 11:**
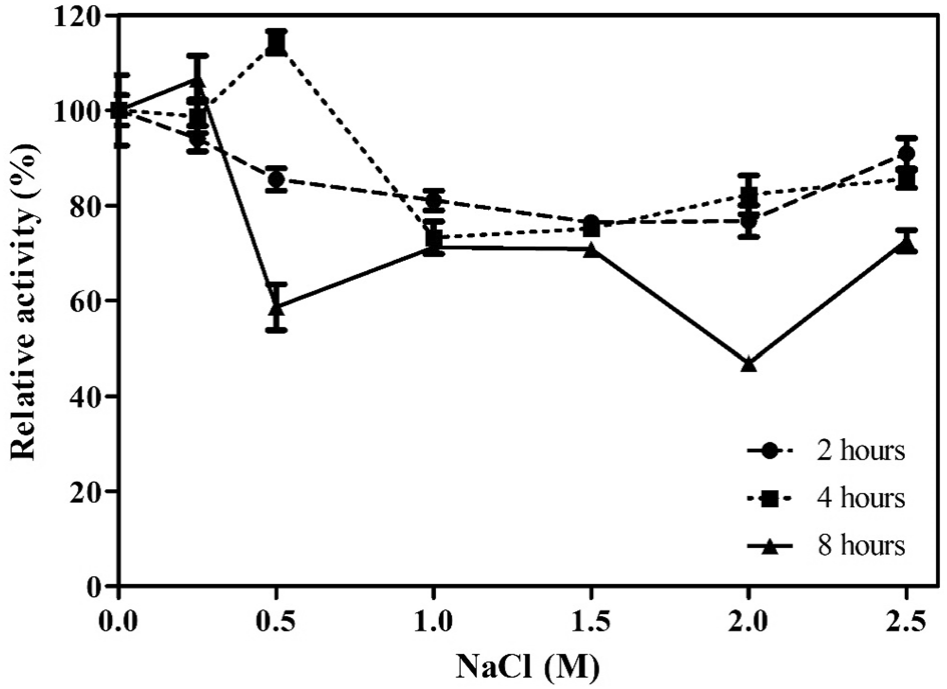
Residual activity after exposure of the enzyme to NaCl at concentrations of 0.1 to 2.5 M for up to 8 h at room temperature. Values are represented as the mean percentage ± standard deviation.

The activity of the XylR enzyme decreased by less than 15% in NaCl concentrations up to 2 M (Fig. 10). Moreover, enzyme activity was inhibited by 20% under exposure to 2.5 M NaCl for up to 4 h, and at least 50% enzymatic activity was maintained with further exposure to this treatment for 8 h (Fig. 11).

### XylR is tolerant to organic solvents

**Figure 12:**
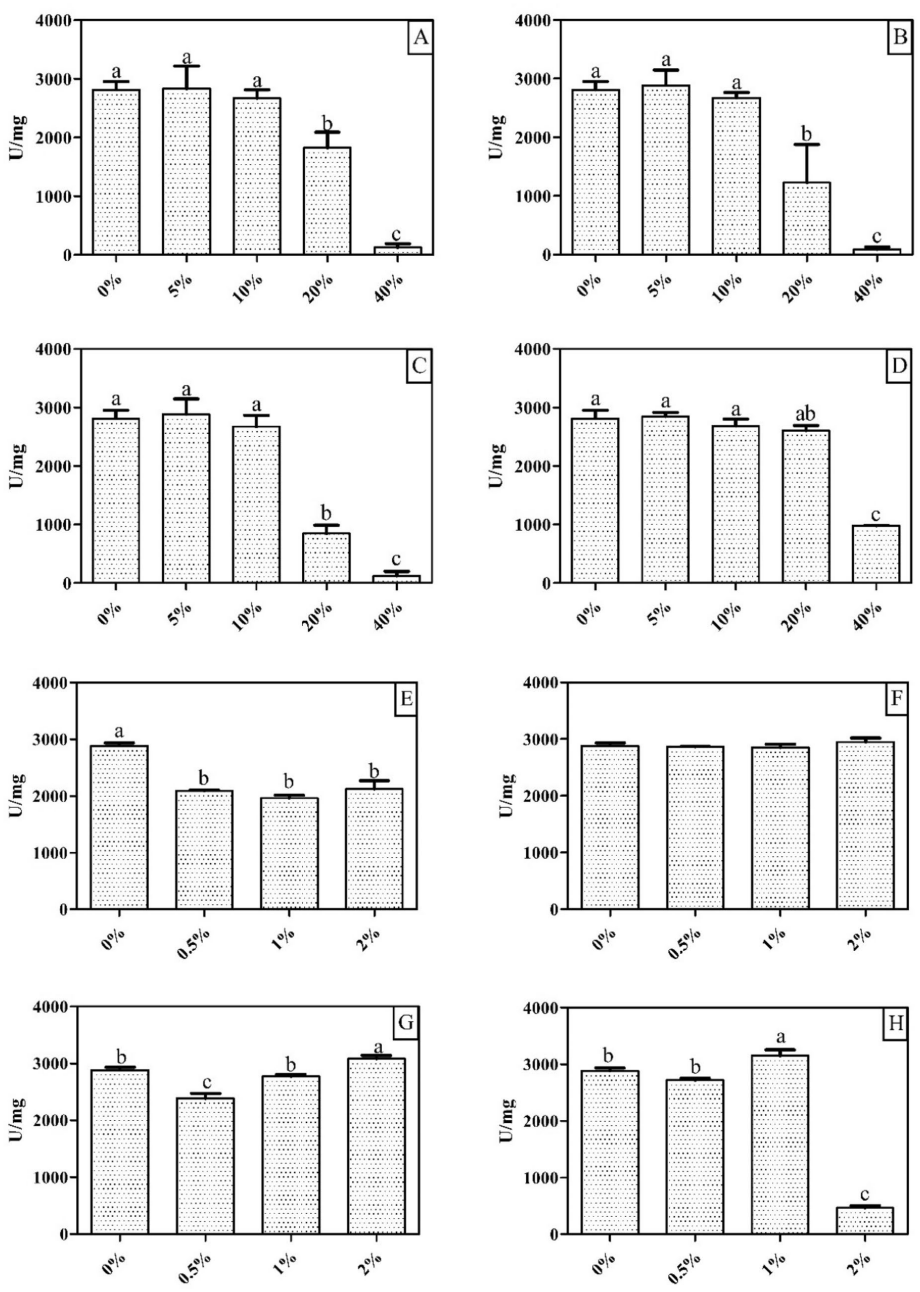
Effect of organic solvents and detergents on XylR activity. (**A**) Methanol; (**B**) Ethanol; (**C**) Propanol; (**D**) Dimethyl Sulfoxide (DMSO); (**E**) Triton X-114; (**F**) Triton X-100; (**G**) Tween20; (**H**) Tween80. The lowercase letters (a, b, c … i) indicate significant differences between each condition tested in the experiment according to the ANOVA and Tukey's test at 5% probability. Values are presented as the mean ± standard error.

The effect of different metal ions and EDTA on XylR activity is shown in Table 2. Enzyme activity was reduced by less than 15% under exposure to Ca^+2^, Mg^+2^ and Mn^+2^ ions and by 60% under exposure to Co^+2^. Moreover, complete inhibition of XylR enzymatic activity was observed with ions Zn^+2^, Hg^+2^ and Cu^+2^. The presence of EDTA did not inhibit the enzyme, whereas the studied organic solvents (methanol, ethanol, propanol, DMSO, Triton X-114, Triton X-100, Tween 20 and Tween 80) caused inhibitory effects on the enzymatic activity only at concentrations above 20% (v/v) (Fig. 12). Additionally, XylR remained stable in the presence of detergents at the studied concentrations except for Tween 80 at 2%, which resulted in 60% inhibition of enzymatic activity.

**Table 2:** Effect of metallic ions, detergents and organic solvents on XylR activity. Values are presented as the mean percentage ± standard deviation; nd = not detected.

Metallic ions (mM)	Relative activity (%)				
	1	2	3	4	5
MnCl_2_	167.8 ± 3.2	161.5 ± 2.8	145.7 ± 11.4	135.8 ± 4.0	137.6 ± 3.0
MgCl_2_	98.0 ± 2.3	100.7 ± 2.7	95.0 ± 0.4	97.8 ± 1.3	89.2 ± 2.9
MgSO_4_	94.1 ± 3.0	91.0 ± 3.9	71.0 ± 16.6	76.7 ± 8.1	88.7 ± 1.7
CaCl_2_	97.5 ± 0.5	99.9 ± 0.8	97.1 ± 2.9	93.9 ± 1.2	95.4 ± 2.0
CoCl_2_	82.4 ± 1.6	74.7 ± 2.5	103.0 ± 0.6	69.0 ± 0.1	39.5 ± 1.2
ZnSO_4_	3.1 ± 2.0	1.3 ± 1.1	Nd	nd	nd
ZnCl_2_	4.7 ± 2.1	Nd	Nd	nd	nd
HgCl_2_	3.5 ± 0.1	4.8 ± 0.7	4.3 ± 0.5	nd	nd
CuSO_4_	0.3 ± 0.1	Nd	Nd	nd	nd
EDTA	107.2 ± 2	106.3 ± 1.5	100.6 ± 2.6	96.7 + 3.2	110.2 ± 3.5

## Discussion

In this study, the XylR enzyme was identified from the rumen metagenome and characterized as a bifunctional xylanase that is tolerant to salt and organic solvents. This enzyme should be a promising candidate for applications in xylan degradation; moreover, its constituent radicals (ferulic acid or p-cumaric acid) are united by ester bonds and can be applied in industrial biotechnological procedures.

The production of recombinant protein from the discovery of new genes has made it possible to leverage the production of lignocellulose-degrading enzymes for the best use of this abundant raw material. Among xylanolitic enzymes, the endo-β-xylanases of the GH10 and GH11 families are more efficient and have greater temperature and pH tolerance^18^. The GH10 family can act on low molecular weight cellulose substrates, such as aryl-cellobiosides, and some cell-oligosaccharides^4^ and thus is a better candidate for the degradation of heterogeneous compounds. Because of the ability of GH10 xylanases to act on short, soluble and branched xylo-oligosaccharides, genes that transcribe these enzymes have been identified in metagenomic databases on organisms that are efficient in hydrolyzing lignocellulose, such as ruminants^11,12,19,20^ and termites^21^. Such work facilitates the search for these types of enzymes and the identification of their hydrolysis mechanisms and structures.

The gene transcribed by *xylr* that was found in the *Nelore cattle* rumen metagenome shared 64% similarity with an endo-1,4-β-xylanase/feruloylesterase from *Prevotella ruminicola*, which is a gram-negative bacterium and one of the most abundant genera in the bovine rumen^22^. The presence of the two glutamate residues (Fig. 1) in the conserved regions _159_WDVVNEA_163_ and _280_TELD_283_ reinforces the catalysis mechanism characteristic of the GH10 family based on the retention of the β configuration of the anomeric carbon^4^ and signals the presence of this enzyme's active site as well as the conservation of these sequences in different species^23,24^. The presence of serine in the conserved GXSXG region (Fig. 2) signals the active site characteristic of carboxylesterases^25^.

XylR cloning was performed without the signal peptide predicted by SignalP 4.0^26^, which optimized the process of obtaining the enzyme from the soluble fraction of *E. coli* BL21 (DE3), which was purified, and heterologous enzyme expression was confirmed by western blot analysis. XylR had endo-β-1,4-xylanase and esterase activities in short chain esters (Fig. 6), which confirms the theoretical predictions of the conserved domains and their bifunctional activity. The kinetic constant values for xylan from beechwood substrate (Table 1) indicate a high efficiency in xylan degradation when compared with those isolated and characterized from species such as *Thermotoga thermarum* (289 mg ml^−1^ s^−1^)^27^, *Remersonia thermophila* CBS 540.69 (417.4 mg ml^−1^ s^−1^)^28^ and *Bacillus* sp. SN5 (142.6 mg ml^−1^ s^−1^)^29^. The affinity for p-nitrofenyl acetate indicates that XylR has sterasic activity and is capable of hydrolyzing small molecules containing ester bonds^30^, although its efficiency was similar to that of previously characterized esterases, such as from the fungus R*hizomucor miehei* (0.37 mM^−1^ s^−1^)^31^ and the bacterium *Bacillus pseudofirmus* OF4 (3.4 µM^−1^ s^−1^)^32^. The specificity for short-chain esterase substrates relative to that of endo-β-1,4-xylanase indicates that this enzyme has an esterase action in addition to that of xylanase, which helps in the digestibility of heteroxylans.

Enzymes with xylanase/esterase bifunctional activity have been previously described^33,34^, and the importance of the synergism between esterase and xylanase activities in hemicellulose degradation has been characterized^35^. The xylan main chain has a high number of ferulic and cumaric acid ester radicals, and the addition of esterases increases their digestibility during bioconversion^13^. The synergistic action of xylanases and esterases is due to the creation of new xylanase binding sites after the cooperative removal of ester radicals between enzymes^36^. In this sense, the enzymatic hydrolysis of ester bonds becomes an important step towards the complete degradation of this type of hemicellulose, which helps to fully utilize lignocellulosic biomass.

The optimal hydrolysis pH of XylR endo-β-1,4-xylanase occurred in 0.1 M sodium acetate buffer at pH 6.5 (Fig. 7), and 50% of its activity was maintained between pH 5.5 and 6.5. This result was similar to that found for endo-β-1,4-xylanases with different origins and biochemical properties, including a thermostable enzyme of *Marasmius* sp. (0.1 M sodium acetate buffer, pH 6.0)^37^; a GH10 family enzyme from *Bacillus subtilis* B10 (0.1 M citrate–phosphate buffer, pH 6.0)^38^ and *Kitasatospora* sp. (0.05 M sodium acetate buffer, pH 6.0)^39^; a salt-resistant strain from *Bacillus subtilis* cho40 (0.05 M citrate buffer, pH 6.0)^40^; and an enzyme obtained from the chicken cecum metagenome (0.05 M sodium phosphate buffer, pH 6.5)^41^. This characteristic reinforces that endo-β-1,4-xylanases activity occurs at acidic to neutral pH values, thus indicating the importance of this range of action in different environments.

The endo-β-1,4-xylanase from XylR exhibited a capacity for hydrolysis at 30 and 45 °C, with optimum hydrolysis occurring at 37 °C (Fig. 8) and stable hydrolysis occurring at 50 °C for one hour (Fig. 10). Hydrolysis activity at room temperature is an important characteristic for the solubilization of hemicelluloses, which would not need thermal control in the range of 30 to 45 °C. This result is similar to that for xylanases obtained from other microorganisms, such as *Paenibacillus xylanilyticus* KJ-03 (40 °C)^42^, *Sorangium cellulosum* So9733-1 (30 °C)^43^, and *Bacillus* sp. SN5 (40 °C)^29^.

The Ca^+2^, Mg^+2^ and Mn^+2^ ions did not inhibit XylR activity, which indicates that this enzyme is tolerant to these metals. Ca^+2^ and Mg^+2^ ions are macronutrients normally found in lignocellulosic biomasses^44^, and stable activity of XylR in the presence of these ions reinforces its potential application in hydrolyzing biomasses of different origins. Additionally, the absence of an inhibitory effect of EDTA indicates that XylR is not a metal-dependent enzyme, which could be an advantage in large-scale biotechnological processes because this enzyme would not require the addition of metal ions for enzymatic catalysis, thereby reducing the operational cost. However, the metallic ions Zn^+2^, Cu^+2^ and Hg^+2^ caused total inhibition of XylR activity at low concentrations (Table 2); therefore, these ions should be avoided in biotechnological procedures using this enzyme. The presence of Cu^+2^-inhibiting xylanases has been described in previous studies^39,45^.

The tolerance of XylR to 2 M NaCl in the reaction medium (Fig. 10) and 2.5 M for up to 8 h (Fig. 11) indicates an important characteristic of this enzyme, thus demonstrating its feasibility for use in the biotechnological sector. Halotolerant xylanases have been previously reported^40,46,47^ as feed additives for broilers^48^ and other farm animals because the diets of these animals are routinely formulated with the inclusion of NaCl; moreover, they are included in pretreatments containing high concentrations of NaCl, such as for cellulose solubilization and depolymerization^49^, because they increase the efficiency and synergy between these pretreatments. Additionally, the tolerance of XylR to organic solvents (Fig. 12), such as methanol, ethanol and propanol, at concentrations up to 20% (v/v) increases the range of biotechnological processes to which it can be applied, such as the continuous production of lignocellulosic ethanol, in which the saccharification and fermentation steps occur in the same bioreactor^50^. Interactions between the hydrophobic ends of a protein and organic solvents can affect an enzyme’s stability; however, XylR showed a low degree of hydrophobicity, which corroborates its resistance to the tested organic solvents.

Xylanases are used in various industrial processes, such as second-generation ethanol production processes^3^, xylooligosaccharide production^8^ and xylitol acquisition^9^. To use lignocellulosic biomass as a raw material, different treatments are required for its solubilization. Currently, efficient treatments to facilitate the use of this material are generally based on chemical compounds that cause environmental contamination^2^; thus, xylanases has been used for the depolymerization of hemicellulose in biotechnological processes.

## Conclusions and future perspectives

In conclusion, data mining of the rumen metagenome identified XylR as a bifunctional enzyme xylanase/esterase that is tolerant to NaCl and organic solvents, thus indicating the diversity of biomolecules that can be exploited in these environments to overcome current barriers for the production of lignocellulosic biomass. Here, we demonstrate that XylR is able to more efficiently degrade the xylan skeleton and its radical esters in biomasses with a high hemicellulose content. The tolerance of this enzyme to high concentrations of salt makes indicates its versatility and suitability for pretreatments that usually require high concentrations of salts. The use of XylR could extend the composition of enzymatic cocktails for the production of lignocellulosic ethanol and food additives in animal feeds and lead to more efficient production of xylooligosaccharides.

In future research, tests will be performed to assay the efficiency of this enzyme in the hydrolysis of lignocellulosic biomass using agricultural waste (e.g., straw and sugarcane bagasse) as raw material and in pretreatments similar to those used in industry to determine its practical applicability. To further understand its structure, X-ray crystallography analysis should be performed to determine its bifunctional activity and its active sites. Additionally, this analysis would help to provide new insights about the behavior of this enzyme under high concentrations of NaCl.

## Material and methods

### Bioinformatics analysis

The functions of the xylanase sequence were inferred based on its similarity to the reference sequences of endo-β-1,4-xylanase from the GH10 family, which was extracted from the American database of the National Center for Biotechnology Information (NCBI), and the sequence was then submitted to the database eggNOG orthologists^51^. Prospecting was performed using the metagenomic database of the Laboratory of Biochemistry and Plant Microorganisms (LBMP) and the Illumina HiScanSQ sequencing data set from *Nelore cattle* rumen (public data access: SRX818104). ORFs (open reading frames) were selected with an e-value of -30, aligned with CLUSTALW^52^ and verified for enzymatic domains in the Pfam database^53^. The sequences were screened based on predictions in silico in terms of the conserved domains^54^, presence of signal peptides^26^ and similarity to sequences others available at the NCBI^15^. The secondary structure prediction was performed based on multiple alignment of the sequences by the Clustal Omega tool^16^ (https://www.ebi.ac.uk/Tools/msa/clustalo/), and assembly and prediction was performed using EsPript 3.0^17^.

### Construction of the recombinant vector

The plasmids were prepared by amplifying the gene encoding the metagenomic DNA of the samples extracted for the sequences available in the LBMP^55^. The following specific primers (Sigma-Aldrich) were synthesized using the commercial PCRBIO Ultra Mix 2 × Kit^26^: forward 5'-TATAgaattcTTCGGACGCAATCCAGACACCAATCC-3', which contains the restriction site *Eco*RI, and reverse 3'-TATAaagcttTTACTTGAACAGCAATTGAG-5', which contains the restriction sites *Eco*RI and *Hind*III. The fragment corresponding to the gene was purified by the Zymoclean Gel DNA Recovery Kit from ZymoResearch. The vector pET28a ( +) (INVITROGEN) and insert were restricted with restriction enzymes (FastDigest EcoRI, Thermo Scientific; and FastDigest *Hind*III, Thermo Scientific) and dephosphorylated with the enzyme Fast Alkaline Phosphatase (1 U/µL, Thermo Scientific). The connection between the insert and the vector was performed according to the protocol of Sambrook and Russel^56^ using the T4 DNA ligase enzyme (New England Biolabs) in a 3:1 ratio (insert: vector).

### Transformation and confirmation of cloning

Transformation into competent *E. coli* BL21 (DE3) cells and positive clone selection were performed on a solid LB plate containing 50 mg/mL kanamycin. For the positive clones, plasmid DNA was extracted by the Wizard Plus SV Kit minipreps DNA purification System (Promega) and the presence of the gene was verified by conventional PCR and Sanger sequencing. The sequencing of the positive clones was performed on an ABI 3130xl platform, and the sequencing reaction protocol followed that described by the manufacturer of the BigDye Terminator v3.1 Cycle Sequencing Kit and used the T7 forward and T7 reverse primers. The procedures were performed by Dr. Camila Cesário Fernandes at Universidade Estadual Paulista—UNESP, Department of Technology, Centralized Multi-User DNA Sequencing Laboratory and Gene Expression Analysis—LMSeq for sequencing (Process FAPESP: 2009/53984-2). The results of the forward and reverse sequences for each gene were analyzed using the BioEdit Sequence Alignment Editor version 7.2.5^57^ through ClustalW^52^ alignment between the contigs and the reference gene obtained from the metagenome.

### Expression and purification of the heterologous protein

XylR was expressed in LB pH 7.0 medium (for 1 L: 10.0 g of tryptone, 5.0 g of yeast extract and 10.0 g of NaCl) containing 50 mg/mL kanamycin and induced with 0.1 mM isopropyl β-D-1-thiogalactopyranoside (IPTG) after reaching an OD_600_ of 0.4–0.6 at 30 °C for 22 h. The enzyme was extracted in 20 mM Tris HCl buffer (pH 7.5) containing 100 mM NaCl and 10% glycerol and treated with 1 mg/mL lysozyme in an ice bath for 1 h. The cells were ruptured by ultrasound with a Branson Sonifier 250 sonicator and centrifuged at 10,000 g at 4 °C to obtain soluble extract. Purification was performed by affinity chromatography for immobilized metal ions in Ni–NTA resin (Qiagen, Hilden, Germany), eluted in the 100 mM imidazole fraction and subjected to Hiload 16/600 Superdex 200 column gel filtration (GE Healthcare Bio-Sciences, Uppsala, Sweden) using the ÄKTA pure chromatography system (GE Healthcare, USA). Elution was performed at a flow of 0.5 ml/minute in 20 mM Tris HCl pH 7.5 buffer containing 200 mM NaCl and 5% glycerol.

### Biochemical characteristics of XylR

#### Polyacrylamide gel electrophoresis in the presence of SDS (SDS-PAGE)

Protein samples were analyzed by denaturing electrophoresis on SDS-PAGE polyacrylamide gels^58^. The samples were previously incubated at 100 °C for five minutes in sample buffer (62 mM Tris HCl (pH 6.8) containing 20% glycerol, 4% SDS, 5% β-mercaptoethanol and 0.02% bromophenol blue) and applied in a 10% polyacrylamide gel containing SDS. Protein separation was carried out by applying an electric field (100 V) for 2 h. The gels were stained using the Coomassie Blue method (0.2% Coomassie Brilliant blue, 40% methanol, and 10% acetic acid).

#### Western blot analysis

The 10% SDS polyacrylamide gel containing the protein sample was transferred to a polyvinylidene difluoride (PVDF, Thermo Fisher Scientific) membrane with a porosity of 0.45 µm using Mini Trans-Blot (Bio-Rad Laboratories, Hercules, CA, USA) in 10 mM pH 11.0 CAPS buffer containing 10% methanol at 90 V for 45 min at 4 °C. Nonspecific sites were blocked with phosphate-buffered saline (PBS), with Tween 20 (0.02%) and 5% dry fat-free milk (dilution 1: 1000). The membrane was incubated with the anti-polyhistidine monoclonal antibody (H1029, Sigma, Saint Louis, MO) and the secondary peroxidase-conjugated anti-mouse IgG antibody (A9044, Sigma, Saint Louis, MO). Development was carried out in the presence of 3,3′-diaminobenzidine tetrahydrochloride in 15 ml PBS (pH 7.6) containing 12 μl of 30% H2O2.

#### Zymogram

Electrophoresis was performed on a polyacrylamide gel (6%) copolymerized with 1% xylan from beechwood in the absence of SDS under 100 V for 2 h. The sample was previously diluted in a 1:1 ratio with 62 mM Tris HCl buffer (pH 6.8) containing 20% glycerol and 0.02% bromophenol blue. The gel was incubated at 37 °C in 0.10 M sodium acetate buffer at pH 6.5 for 1 h and then stained with 0.1% Congo red.

#### Determination of protein concentration

The protein concentration was determined using a commercial Bio-Rad kit based on the method proposed by Bradford^59^ using serum albumin as a protein standard.

#### Determination of xylanase activity

Xylanase activity was determined by adding 20 µL of the enzyme at 0.1 mg/mL to 0.10 M sodium acetate buffer (pH 6.5) containing 0.2% (w/v) xylan from beechwood (Meganzyme) for 15 min at 37 °C. Reducing sugars were quantified using the 1–3-dinitrosalicylic acid (DNS) method^60^, and reading was performed on a spectrophotometer at 540 nm. One unit of enzymatic activity was defined as 1 µmol of reducing sugar per minute per mg of protein under the previously established standard test conditions. The tests were carried out in triplicate, and each reaction included a control without the enzyme to measure the spontaneous hydrolysis of the substrate.

#### Effect of temperature and pH

The optimum pH for xylanase activity was determined using 0.1 M sodium acetate (pH 3.0–6.5), 0.1 M Tris HCl (pH 6.5–7.5) and 0.1 M AMPOL (pH 8.5–10). The optimum temperature was determined by placing the enzyme in 0.1 M sodium acetate buffer at different temperatures (4–95 °C) for 15 min. The parameters km and Vmax were determined using xylan from beechwood at concentrations of 0.025 to 5 mg/mL in 0.1 M sodium acetate buffer (pH 6.5).

#### Effect of metal ions

The influence of the ions Co^+2^, Cu^+2^, Hg^+2^, Mg^+2^, Mn^+2^, and Zn^+2^ on the activity of xylanase was evaluated at concentrations of 1, 2, 3, 4 and 5 mM in the reaction medium. The relative activity was calculated from the control of each test, which did not include the addition of ions (100%).

#### Effect of sodium chloride

The effect of NaCl on xylanase activity was evaluated by adding NaCl at concentrations of 0.1 to 2 M to the reaction medium and exposing the enzyme to NaCl at concentrations of 0.1 to 2.5 M for 2, 4 and 8 h. Relative activity was calculated by adding to each assay a control that did not include the addition of NaCl (100%).

#### Effect of temperature

The effect of temperature on xylanase activity was determined by preincubation of XylR in the absence of the substrate at 45, 50, 55 and 60 °C for 15, 30, 45 and 60 min. After incubation, the activity was determined at 37 °C and the residual percentage was estimated.

#### Determination of esterase activity

The esterase activity was determined discontinuously in a spectrophotometer at 405 nm. The reaction was initiated by adding 20 µL of the enzyme at 0.1 mg/mL to 0.1 M sodium acetate buffer at pH 6.5 and 1 mM p-nitrophenyl acetate for 15 min at 37 °C. Substrate affinity was determined using the following substrates (1 mM, Sigma-Aldrich): *p*-nitrofenyl acetate, *p*-nitrofenyl butyrate, *p*-nitrofenyl valerate, *p*-nitrofenyl octanoate, *p*-nitrofenyl dodecanoate, *p*-nitrofenyl myristate, and *p*-nitrofenyl palmitate. One unit of enzymatic activity was defined as 1 µmol *p*-nitrophenol released per minute per mg of protein under previously established standard assay conditions. The tests were performed in triplicate, and each reaction included a control without the enzyme to measure the spontaneous hydrolysis of the substrate.

#### Determination of kinetic parameters

The catalytic parameters K_m_ (Michaelis–Menten constant), V_ma_x (maximum reaction speed), kcat (catalytic constant) and kcat. K_m_^−1^ (catalytic efficiency) was determined for endo-β-1,4-xylanase activity with xylan from beechwood as the substrate, and the concentration varied from 0.025 to 2.5 mg/mL. For esterase activity, *p*-nitrofenyl acetate was used as the substrate, and the concentration varied from 0.75 to 5 mM. The data were tested using the F test (p < 0.05) to determine the best kinetic model: H_0_ = Michaelis–Menten and H_1_ = sigmoidal. The normality of the residues was verified by the Shapiro–Wilk W test. The hypothesis test and nonlinear regression of the data by the Michaelis–Menten equation were performed using GraphPad Prism Software, version 5.00 for Windows (GraphPad Software, San Diego, California, USA).

## Supplementary Information

Supplementary Information.
