# Supplementary material for: Characterization of a new bifunctional endo-1,4-β-xylanase/esterase found in the rumen metagenome
Source: Sci Rep. 2021 May 17;11:10440. doi: 10.1038/s41598-021-89916-8 (PMC8128909; doi:10.1038/s41598-021-89916-8)
Supplement: Supplementary file 1 — Supplementary Information. [file 41598_2021_89916_MOESM1_ESM.docx]

**Characterization of a new bifunctional endo-1,4-β-xylanase/esterase found in the rumen metagenome**

Gabriella Cavazzini Pavarina^1,2^, Eliana Gertrudes de Macedo Lemos^1,3^, Natália Sarmanho Monteiro Lima^1,2^, João Martins Pizauro Junior^1*^

^1^ Technology department, Sao Paulo State Univerversity (Unesp), Faculty of Agricultural and Veterinary Sciences. Via de Acesso Prof. Paulo Donato Castellane S/N, km 5, São Paulo Brazil.

^2^ Graduate student of Agricultural Microbiology, Jaboticabal, Sao Paulo, Brasil.

^3^ Molecular Biology Laboratory, Bioenergy Research Institute (IPBEN), Unesp – Jaboticabal, Sao Paulo, Brazil.

^*^Correspondence and requests for materials should be addressed to J. M. P. Jr (e-mail: j.pizauro@unesp.br)

**Supplementary Information**

| ***NCBI Collections*** | **XylR** |
| --- | --- |
| *non-redundant* | 74% similarity with the hypothetical protein of the GH10 family of the organism *Bacteroidales bacterium*, query cover 99%  Number of access: PWL58549.1 |
| *Swissprot* | 64% similarity with the endo-1,4-β-xylanase/ feruloylesterase enzyme from *Prevotella ruminicola* 23, *query cover* de 99%  Number of access: D5EY13.1 |
| *Pat* | 66% similarity and 99% de *query cover* with a US patent.  Number of access: AKY00390.1 |

**Table S1.** Similarity analysis of the XylR amino acid sequence to the NCBI available collections.


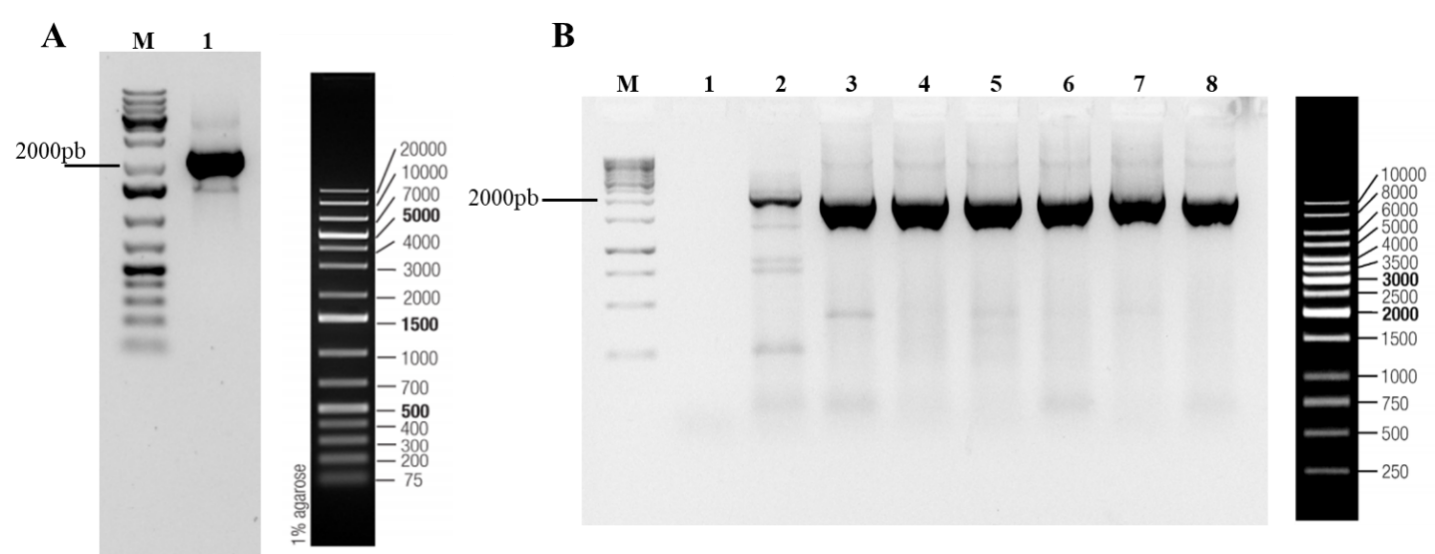


**Figure S1. (A)** 1,5% agarose gel containing the amplified and purified xylr gene (2100pb).
Legend: (M) Gene Ruler marker 1Kb DNA ladder; (1) xylr gene cloned and amplified. **(B)** 1,5% agarose gel containing E. coli BL21(DE3) positive clones containing the xylr gene. Legend: (M) Gene Ruler marker 1Kb DNA ladder; (1) Negative control; (2) Positive control; (3) Clone 1; (4) Clone 2; (5) Clone 3; (6) Clone 4; (7) Clone 5; (8) Clone 6.


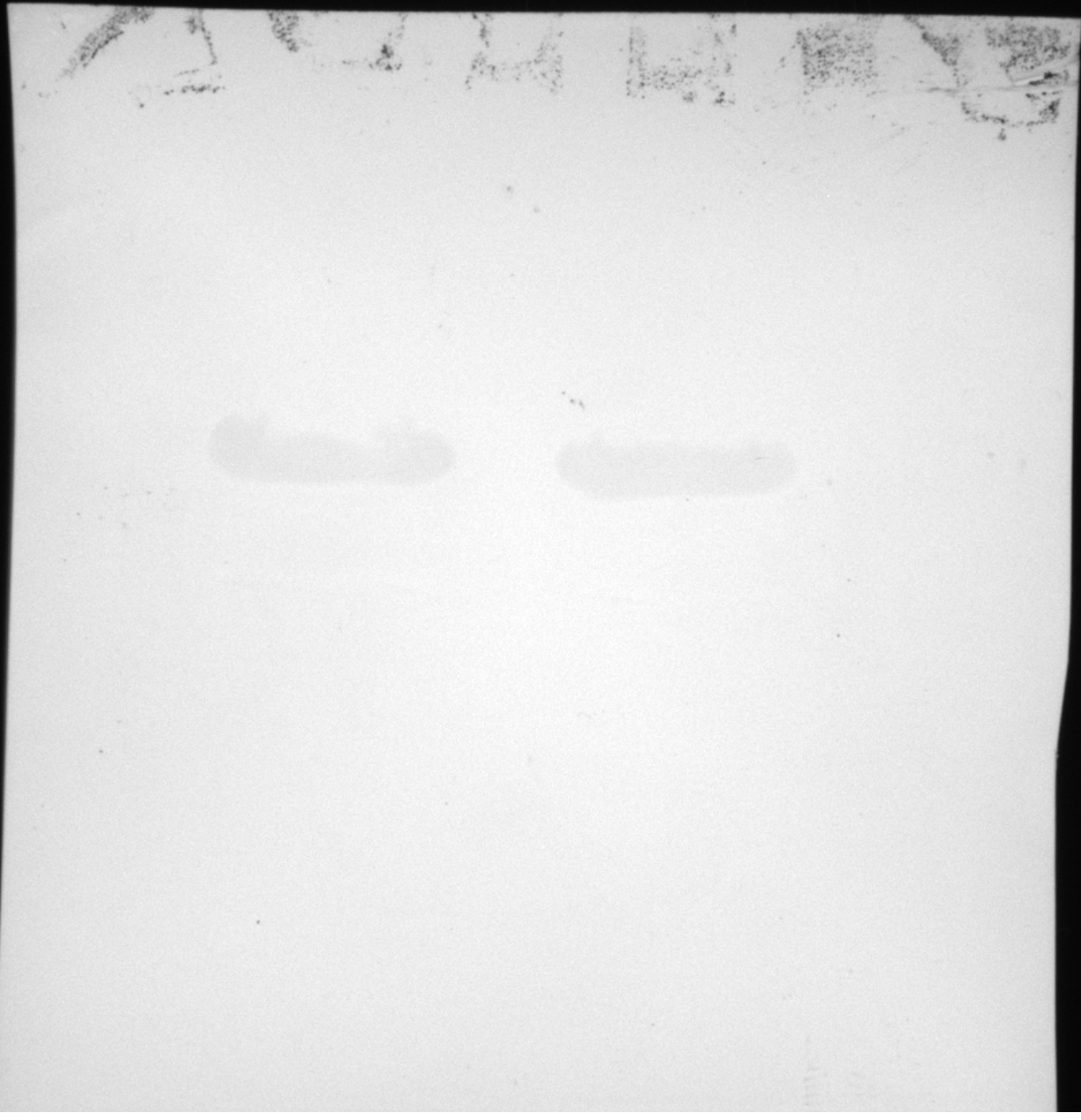


**Figure S2.** Western blot of the purified enzyme using anti-His_6_ antibodies.
